# The Prospering of Macromolecular Materials Based on Plant Oils within the Blooming Field of Polymers from Renewable Resources †

**DOI:** 10.3390/polym13111722

**Published:** 2021-05-25

**Authors:** Julio Antonio Conti Silva, Luan Moreira Grilo, Alessandro Gandini, Talita Martins Lacerda

**Affiliations:** 1 Biotechnology Department, Lorena School of Engineering, University of São Paulo, CEP 12602-810 Lorena, SP, Brazil; julioconti@usp.br (J.A.C.S.); luanmgrilo@gmail.com (L.M.G.); 2 Graduate School of Engineering in Paper, Print Media and Biomaterials (Grenoble INP-Pagora), University Grenoble Alpes, LGP2, CEDEX 9, 38402 Saint Martin d’Hères, France; agandini@iqsc.usp.br

**Keywords:** polymers from renewable resources, vegetable oils, triglycerides, fatty acids, copolymerization, tung oil

## Abstract

This paper provides an overview of the recent progress in research and development dealing with polymers derived from plant oils. It highlights the widening interest in novel approaches to the synthesis, characterization, and properties of these materials from renewable resources and emphasizes their growing impact on sustainable macromolecular science and technology. The monomers used include unmodified triglycerides, their fatty acids or the corresponding esters, and chemically modified triglycerides and fatty acid esters. Comonomers include styrene, divinylbenzene, acrylics, furan derivatives, epoxides, etc. The synthetic pathways adopted for the preparation of these materials are very varied, going from traditional free radical and cationic polymerizations to polycondensation reactions, as well as metatheses and Diels–Alder syntheses. In addition to this general appraisal, the specific topic of the use of tung oil as a source of original polymers, copolymers, and (nano)composites is discussed in greater detail in terms of mechanisms, structures, properties, and possible applications.

## 1. Introduction

The presence of polymers in all sectors of today’s society is irreversible and has generated debate about the harmful consequences that they cause to the environment, such as greenhouse gas emissions and the production of incalculable amounts of waste. In this context, efforts have been directed, in both academic and industrial sectors, to the development of alternative renewable materials [1,2,3], with priority to those that have also potential to be recovered after use. The application of biological platforms for the synthesis of macromolecular materials is a very promising strategy, as it opens the way for the production of original polymers with reduced environmental impact. The development of new technologies for the production of macromolecular materials from renewable resources is now boosted by a widespread discussion on how polymers should advance in a sustainable society based on a circular economy, considering environmental aspects at the same time as promoting an efficient conversion of resources and correct and reduced disposal of waste [4]. Monomers prepared from vegetable biomass can produce versatile polymers, and many of them are based on consolidated technologies or on those under advanced development, which is the case of biobased ethylene, ethylene glycol, and terephthalic acid [5].

**Figure 1:**
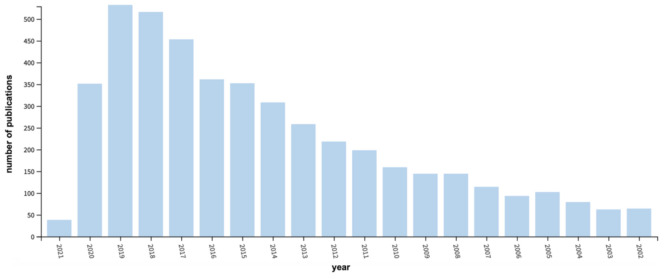
Number of yearly publications on polymers from vegetable oils. Data from www.webofknowledge.com (accessed on 9 March 2021) [14].

**Figure 2:**
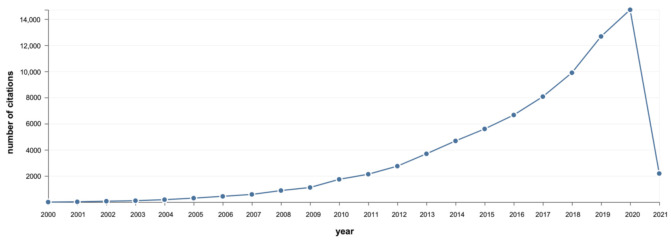
Number of yearly citations on polymers from vegetable oils. Data from www.webofknowledge.com (accessed on 9 March 2021) [14].

Vegetable oils constitute one of the most abundant varieties of renewable resources on Earth and have been widely exploited by humans for food and feed, energy generation, and as precursors for a multitude of products and materials. A full understanding of their chemical structure and their reactivity has widened the range of applications of these valuable raw materials during the second half of the 20th century, meeting the polymer revolution that occurred simultaneously. Vegetable oils and their derivatives are the protagonists for the development of polymers from biomass [6,7,8,9,10,11,12,13]. Indeed, an exponential behavior is observed when both the number of scientific publications using the keywords “polymers” and “vegetable oils” and their corresponding citations are plotted as a function of time considering only the last two decades ( Figure 1; Figure 2), which is a convincing demonstration that the subject is far from being fully exploited.

To pursue novel polymers from vegetable oils, different approaches are possible, including, not exhaustively, the breakdown of triglycerides by transesterification processes and the chemical modification of the fatty acids’ double bonds by means of metathesis, epoxidation, and click reactions [6]. The employment of alternative strategies to established methodologies, such as the use of enzymatic catalysis for the modification and polymerization of vegetable oils and their derivatives, has been slowly growing over the past few years [15,16,17]. The various possibilities in this regard have led to the production of polymeric materials with interesting properties and promising applications, such as thermosets, linear polyesters, polyurethanes, polyamides, and their blends and composites, among others.

The major commercial applications of vegetable oils are related to the food industry. However, over the past twenty years, significant growth in the proportion of non-food applications of the most common vegetable oils has been observed [18], with the polymer industry contributing largely to this trend. In fact, vegetable oils have been used to produce polymers for millennia. The so-called drying oils, such as linseed and tung oils, were extensively exploited before synthetic epoxy and urethane resins began to replace them during the 1950’s for the production of waterproof films [19]. Vegetable oils are also considered on the industrial scale for the preparation of polymer additives (e.g., epoxidized soybean oil, often used as a plasticizer) and monomers (e.g., dicarboxylic acids for the synthesis of polyesters or polyamides via polycondensation mechanisms) [20]. The latter possibility includes sebacic acid, which is obtained by the alkaline cleavage of castor oil, and azelaic acid, produced from the oxidation of oleic acid through ozonolysis. These dicarboxylic acids impart high impact strength, hydrolytic stability, hydrophobicity, lower glass transition temperatures, and flexibility to the final materials [18]. In addition, technologies for the commercialization of other dicarboxylic acids derived from vegetable oils, such as adipic acid and dodecanedioic acid, are currently under development by some companies [21].

This monograph aims to provide an up-to-date overview of some important research strategies on the preparation of polymers from plant oils following a recent book on the topic [6], with a particular focus on novel synthetic approaches, as well as on original properties of the ensuing materials. First, the discussion focuses on the use of unmodified triglycerides as monomers, leading, on the one hand, to highly branched or crosslinked soft materials, or, on the other hand, to highly branched or crosslinked materials of higher glass transition temperature (T_g_) via their association with comonomers including styrene, divinylbenzene, acrylics, furan derivatives, etc. Then, attention is directed to the polymerization of fatty acids and the corresponding esters, and of other molecular structures derived from them. A further section is dedicated to the most recent contributions related to composites made of vegetable oil-based continuous phases, which was addressed a few years ago in the monograph published by Zhang et al. [22] Finally, the specific use of tung oil and its main fatty acid as a source of original polymers, copolymers, and nanocomposites is discussed in greater detail in terms of mechanisms, structures, properties, and possible applications. It is important to mention here a very recent contribution by Biermann and collaborators [23], reviewing the use of fatty acids and their derivatives as a source of low molecular weight products, such as fatty alcohols, ethers, amines, among others, and polymers. The authors carefully presented the synthetic routes involved in the preparation of fatty acid-based commodities, including a final section on enzymatic and microbial transformations. Herein, an extended version of a paper published in The First International Conference on “Green” Polymer Materials 2020 is presented [24], including a set of other relevant contributions published on the field of plant oil-based polymers.

## 2. Vegetable Oils: A Unique Source of Chemicals and Monomers

Mostly extracted from the seeds of some species of annual plants, vegetable oils are yellowish viscous liquids at room temperature. According to the United States Department of Agriculture, the total production of major oilseeds around the world in 2019/2020 overcame 570 Mt, with most of them consisting only of soybean. The global 2020/21 oilseed production is forecast at 595 Mt [25]. In Table 1, the world supply and distribution of major oilseeds are presented, comparing the values of 2002/2003 and of 2019/2020.

**Figure 3:**
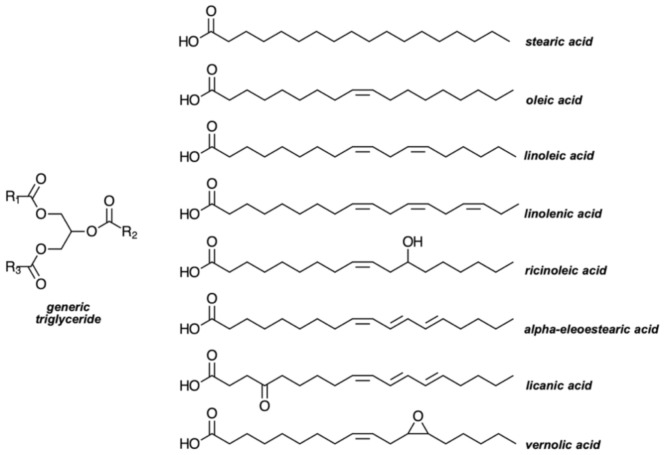
Molecular structure of a generic triglyceride and common fatty acids present in the composition of vegetable oils.

Oil extraction is a critical step, directly impacting the quality and quantity of commercial vegetable oils. Nde and Foncha [26] reviewed some of the most frequently used extraction methods, as well as the optimized conditions often considered, which enhance the yield and the quality of the oil at the same time as reducing the final costs of the whole process. The precise composition of a given vegetable oil, on the other hand, depends on the source from which it has been extracted and on a multitude of edaphoclimatic factors. However, all of them possess a common structure of aliphatic triglycerides, in which the fatty acid chains R_1_, R_2_, and R_3_ are most often identical, but can also vary within a given molecule (Figure 3). Up to the present, more than 1000 fatty acids have been identified, but only about 20 are found in appreciable quantities in vegetable oils [27,28]. Linseed oil, for example, consists mainly of linoleic and linolenic acid, while in castor oil and tung oil, the most abundant fatty acids are, respectively, ricinoleic and α-eleoestearic acid [6,29]. The fatty acid chain may be made of 14–22 carbon atoms, but most members bear 16 or 18 units. The other important feature is the possible presence of C=C unsaturations per molecule, which ranges from 0 to 3. The stereochemistry of the double bonds, the degree of unsaturation, and the length of the fatty acid chains are the main characteristics that determine the physicochemical properties of these oils. In most vegetable oils, the double bonds of the fatty acid chains are in the *cis* configuration (e.g., oleic and linoleic acids), although *trans* counterparts may also be present (e.g., α-eleoestearic and licanic acids). The double bonds are more often non-conjugated (e.g., linoleic and linolenic motifs), but conjugated sequences are also possible (e.g., eleoestearic and licanic structures). Some oils contain fatty acid esters with other moieties along their chains, such as ricinoleic, vernolic, and licanic structures with hydroxyl, epoxy and carbonyl groups, respectively, and others bear fully saturated carbon sequences, as is the case of lauric, myristic, palmitic, and stearic acids [6].

The average degree of unsaturation, measured by the iodine value (i.e., the amount in mg of iodine that reacts with the double bonds of 100 g of a given oil), is particularly relevant in the present context, since the double bonds are the promoters of most of the functionalization, crosslinking and polymerization reactions. Iodine values of some common fatty acids and their triglycerides are given in Table 2. In general, oils are classified as drying if their iodine value is higher than 130, semi-drying if it is between 90 and 130, and non-drying if it is lower than 90 [6]. In a few words, the “drying” process of unsaturated vegetable oils, i.e., their ability to produce film coatings when exposed to air (often termed “siccativity”), consists of a free-radical polymerization mechanism (often referred as oxydopolymerization), in which the oxygen molecule inserts itself into the C–H bonds next to C=C unsaturations to form a hydroperoxide moiety [6]. Therefore, the degree of unsaturation of a given oil determines the rate at which it will “dry”.

**Figure 4:**
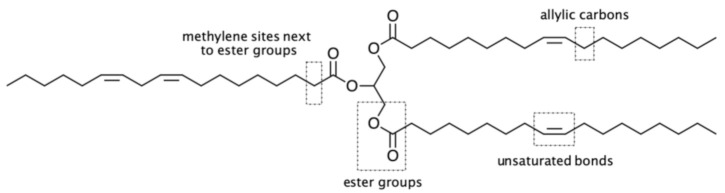
Four main reactive sites of unsaturated triglycerides.

Considering the generic structure of a triglyceride incorporating C=C unsaturations, four sites are susceptible to undergoing reactions or chemical modifications, as shown in Figure 4, with more specific oils bearing other functional groups offering additional sources of chemical exploitation. The large number of naturally available oils and the wide array of possible chemical modifications that can be performed are therefore the keys that allow researchers to continuously achieve a myriad of fascinating molecular structures based on triglycerides. In fact, oils and fats of vegetable and animal origin make up the greatest proportion of the consumption of renewable raw materials in the chemical industry [23,30]. A widely accepted notion asserts that the fossil-based chemical industry is perfectly consolidated—in terms of technology, investments, and profits—to provide the commodities, fine chemicals, and specialties on which our modern society stands [31]. In order to dispel this “dogma”, some obstacles need to be addressed for a wider and more competitive production of chemicals from vegetable oils, and more efficient processes are continuously being designed for this purpose.

In industrial processing, vegetable oils are converted to glycerol plus fatty acids, methyl esters, and fatty alcohols, and subsequent chemical modification steps are often conducted in order to obtain the specific chemicals of interest. Those reactions mainly consist of tackling the unsaturated bonds, as in the case of epoxidation, hydroformylation, dimerization, thiol-ene coupling, oxidative cleavage (ozonolysis), olefin metathesis, pericyclic reactions, and radical additions, as well as transition-metal catalyzed and Diels-Alder syntheses to aromatic compounds [6,23,32]. Among the chemicals of interest, one finds non-exhaustively, soaps, surfactants, emollients, pesticide formulations, and lubricants [23,31,32,33,34,35,36]. Rios and coworkers described the synthesis of new lubricant molecules from castor oil by conducting an initial step of esterification of ricinoleic acid with 2-ethylhexanol, followed by epoxidation and ring-opening of the oxirane rings [37]. Additionally, biodiesel (fatty acid methyl esters, FAMEs) has emerged as a viable alternative in terms of renewable fuel and is industrially produced from the basic-catalyzed transesterification of vegetable oils with methanol or ethanol. The most promising and viable technologies related to the field were reviewed [38], where the authors described classic methods such as homogeneous, heterogeneous, and enzyme catalysts, as well as others based on supercritical fluids, microwave, ultrasound and plasma irradiation.

This broad range of possibilities is also convenient to convert plant oils into a large variety of monomers and polymers [3,6]. As a matter of fact, one can define some crude vegetable oils as natural oligomers, and film coatings based exclusively on unsaturated triglycerides have been long used by man in different contexts. This is the case of tung oil, extracted from the seeds of *Vernicia fordii* nuts, which has a millennial history of exploitation in China as a siccative coating for waterproofing, varnishing, paints, lacquers, and inks, thanks to the highly unsaturated structure of its main triglyceride, made up of α-eleostearic fatty-acid motifs incorporating three conjugated double bonds (Figure 3). This is precisely the aforementioned oxidopolymerization reaction, which passes through the formation of radical intermediates, finally reaching a tridimensional network [6].

**Figure 5:**
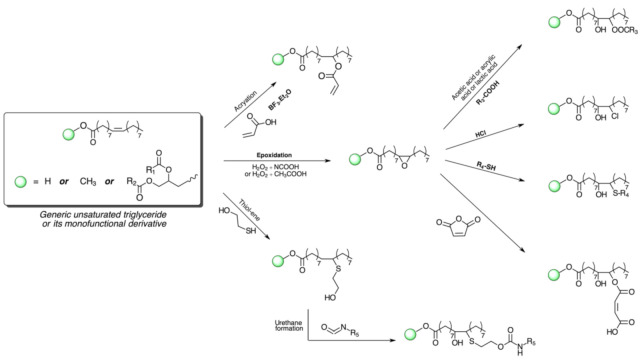
Schematic representation of the most frequent chemical modifications performed on vegetable oils to synthesize monomers and polymers (R_1_ and R_2_ are general fatty acids, as depicted in Figure 3; R_3_, R_4_ and R_5_ are alkyl groups of varying chemical structures).

Since this first empirical exploitation, the strategy of converting plant oils into polymeric materials has vastly evolved, starting with linoleum, developed in the second half of the 19th century. Synthetic polymers based on vegetable oils have already been commercialized on a large scale since the 1950s, with Rilsan^®^, a nylon-11 based on castor oil produced by the French Arkema (Colombes, France) as the main example. From then on, different synthetic routes, controlled processes, and sophisticated catalysts are periodically reported, and today, vegetable oil-based polymeric materials with tunable properties can be designed according to the intended application [6]. Among the possible approaches, one can emphasize the preparation of (i) highly branched and crosslinked materials, resulting from polymerization reactions of polyfunctional triglycerides and (ii) linear materials, obtained by the breakdown of triglycerides into the corresponding fatty acids, which are often chemically modified to be converted into suitable bifunctional monomers (Figure 5). The most relevant contributions related to both strategies are briefly discussed below with particular emphasis on recent advances.

It is important to highlight that, considering the width of the possibilities available, the choice of a specific mechanism or synthetic route for the development of polymers from plant oils is not a simple task, mainly considering an upscaled production. Such analysis depends critically on the raw material used, the extraction methods, the need (or not) for previous purification and chemical modifications, the specific characteristic of polymer synthesis, isolation and properties, and many other parameters. In the end, in order to assess the issues related to the pros and cons of the use of vegetable oils for polymer synthesis, studies must be carried out with respect to technical, economic and, environmental viabilities.

## 3. Branched and Crosslinked Polymers Based on Pristine or Chemically Modified Triglycerides

**Figure 6:**
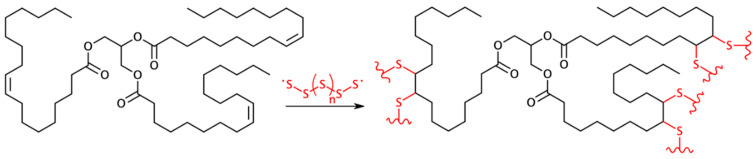
Schematic representation of a polymerization reaction between an oleic acid triglyceride and elemental sulfur. Reproduced with the permission from Ref. [40]. Copyright Sustainable Chemistry and Pharmacy 2019.

The direct polymerization of polyfunctional vegetable oils into thermosets is a possibility that avoids preliminary synthetic steps and therefore represents a more sustainable approach towards polymeric materials [39]. The 1,2-disubstituted C=C unsaturation of common fatty acids often react slowly in the presence of free radical and cationic initiators, and this strategy demands an association with a more reactive comonomer such as styrene and divinylbenzene [6]. A recent and very promising approach is based on the fact that sulfur can act as a comonomer to initiate polymerization when heated to its molten state with unsaturated hydrocarbons. From this reaction has emerged a new class of macromolecular materials called sulfur-based polymers, which are mostly prepared from fossil-based vinyl monomers. Unsaturated vegetable oils may serve as green and viable alternatives in this context, as shown when such bio-based polymers (Figure 6) were indeed synthesized and exhibited good potential for applications as cathode material in batteries such as Li–S batteries, as adsorbents for the removal of mercury and hydrocarbons from polluted water, and as support for smart controlled-release of fertilizers [40,41].

Despite the viability of directly polymerizing crude plant oils, a preliminary treatment for converting triglycerides into more reactive monomers is often more convenient [6]. Recent contributions include the methacrylation of high oleic sunflower oil and incorporation of polyethylene glycol onto its structure. The products were reacted with polyethylene glycol dimethacrylate, and the results indicated that the materials were suitable for drug delivery applications [42]. The acrylation of a palm oil derivative was also reported, which resulted in a branched polymer bearing biodegradable functional groups [43]. A one-step acrylation procedure was applied to palm, olive, peanut, rapeseed, corn, canola, and grapeseed oils, and the products were separately mixed with polyurethane acrylate, trimethylolpropane triacrylate, and a photoinitiator to prepare ultraviolet-curable films [44]. Epoxidation is another suitable preliminary reaction for the insertion of acrylic moieties into the triglyceride backbone, which was the route adopted to prepare acrylated linseed oil and, in a further step, aza-Michael polymers using priamine 1071, amine-terminated poly(propyleneoxide)s (jeffamines), and meta-xylylenediamine as crosslinkers [45]. In a similar vein, acrylated epoxidized soybean oil was copolymerized with methacrylated vanillyl alcohol, which had been synthesized from vanillyl alcohol and methacrylate anhydride, to produce thermoset resins [46].

**Figure 7:**
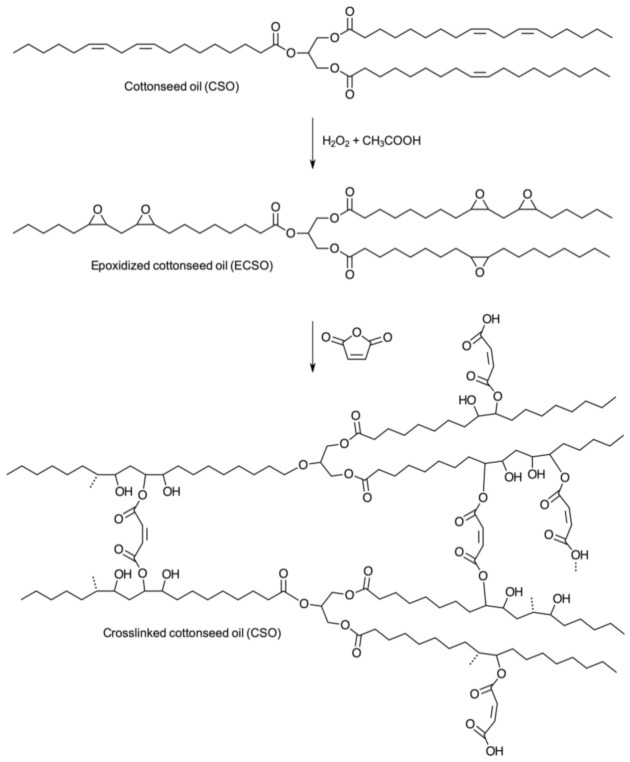
Reaction scheme for the synthesis of crosslinked epoxidized cottonseed oil. Reproduced with the permission from Ref. [48]. Copyright Journal of Applied Polymer Science 2019.

Epoxidized vegetable oils can be also explored for the synthesis of a series of polymers without the need for a preliminary acrylation step. Castor oil was associated with vanillin-furfurylamine-containing benzoxazine and eugenol-furfurylamine-containing benzoxazine resins, to synthesize shape memory polymers [47]. The preparation of crosslinked polymers from epoxidized cottonseed oil and maleic anhydride (Figure 7) resulted in a rigid polymer that could have its mechanical properties tuned by varying the amount of maleic anhydride in the formulation [48]. Epoxidized soybean oil was firstly converted to hydroxylated soybean oil and then employed as a macro-initiator for the ring-opening polymerization of ε-caprolactone using stannous octanoate as a catalyst [49]. Moreover, the effects of incorporating epoxidized vegetable oils (olive, soybean, and linseed) into a thiol-epoxy shape memory network, obtained from gallic acid-based thiol and diglycidyl ether of bisphenol A, were investigated, as the addition of epoxidized vegetable oils reduced the T_g_ and the tensile strength of the resulting polymers, but the elongation at break and toughness were increased up to ten times thanks to the role of epoxidized vegetable oils [50]. The effect of B(C_6_F_5_)_3_ as the initiator for the cationic polymerization of epoxidized fatty acid methyl esters and epoxidized oils with *cis*-disubstituted oxirane groups produced a mixture of linear and cyclic oligomers with molar masses up to 27,000 Da [51].

**Figure 8:**
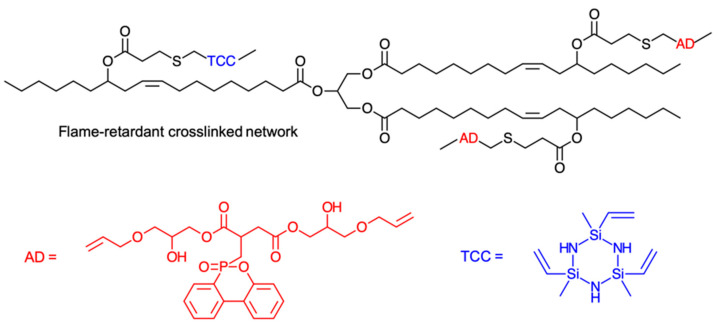
Molecular structure of a flame-retardant crosslinked network made of castor oil, AD and TCC [57].

In another vein, castor oil was submitted to a two-step procedure of maleinization and methacrylation (using, respectively, maleic anhydride and glycidyl methacrylate) to synthesize a highly unsaturated monomer to be further homopolymerized and copolymerized in association with styrene [52]. The final materials were submitted to accelerated weathering tests in order to estimate their long-term performance under environmental conditions. Besides acting as a direct crosslinking agent for crude triglycerides, sulfur can also be useful for the synthesis of plant oil-based reactive monomers, since the thiol-ene click reaction may be applied as a versatile tool to yield high conversions under mild conditions. For instance, the photo-initiated thiol-ene reaction was employed to polymerize rapeseed oil-based multifunctional vinyl oligomers along with thiol monomers, such as pentaerythritol tetrakis (3-mercaptopropionate), pentaerythritol tris (3-mercaptopropionate), and 1,2-ethanedithiol [53]. The thiol-ene reaction, along with the isocyanate polyurethane reaction, was also used to modify soybean oil with urethane branched chains terminated by an epoxy group, producing a bio-based curable resin [54]. In a separate work, the same group then employed the modified soybean oil compounded with acrylates to obtain hybrid resins for stereolithographic 3D printing [55]. A castor oil-based mercaptan, 1,3,5-triethylene-1,3,5-trimethylcyclotriazane (TCC), and a phosphorus-containing flame-retardant molecule (AD) were compounded via photo-initiated thiol-ene click reaction (DMPA photoinitiator under 365 nm light) to create a flame-retardant coating on wood surfaces [56,57] (Figure 8). In a similar way, diethyl allyl phosphonate A was inserted via thiol-ene reaction into a commercial mercaptanized castor oil, and the ensuing polyol was used for the preparation of rigid polyurethanes with different weight percentages of phosphorus and with promising flame retardancy properties, ensuring fire safety for construction and household applications [58].

**Figure 9:**
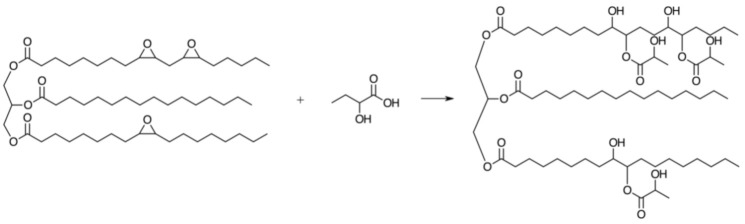
Schematic of the ring-opening reaction between epoxidized soybean oil and lactic acid. Reproduced with the permission from Ref. [68]. Copyright Journal of Applied Polymer Science 2019.

In fact, the preparation of polyurethanes from vegetable oils is a subject that has attracted further attention, since its first report in the literature in the early 1990s [59], related to the preparation of polyurethane foams by mixing waste oil and polypropylene glycol with diphenylmethane diisocyanate, and opened the way to hundreds of other publications belonging to the same field. Interesting and thorough monographs tackling the most relevant advances were recently published, calling upon aspects of synthesis, mechanical and thermal properties of (i) polyurethanes from seed oil-based polyols [60], (ii) dimer fatty acid-based polyols [61], (iii) conductive polyurethanes based on castor oil-based polyols and carbon fiber powder [62], (iv) opportunities and challenges of bio-based polyurethanes with flame retardancy [63] and (v) plant oil-based waterborne polyurethanes [9], viz. ionic and non-ionic polyurethane dispersions prepared in aqueous media. In this context, castor oil displays one key advantage over other plant oils, namely its inherent polyol character for polyurethane synthesis. A waterborne polyurethane network was synthesized using castor oil, dimethylolbutanoic acid, and isophorone diisocyanate and its structural strength could be tailored by the incorporation of different amounts of octahydro-2,5-pentalenediol [64]. Furthermore, the same research group prepared high strength self-healing multi-shape memory waterborne polyurethane networks by introducing controlled amounts of dithiodiphenylamine to the castor oil-based polymer chains [65]. The use of castor oil as a polyol in combination with the previously mentioned sulfur crosslinking strategy was employed to produce high strength vulcanized polyurethane interpenetrating polymer networks. Thus, castor oil was vulcanized with sulfur and subsequently polymerized with polymeric diphenylmethane diisocyanate, forming a coating for the slow release of a urea-based fertilizer [66]. Various synthetic tools can be employed for the production of polyols from other unsaturated vegetable oils. 2-mercaptoethanol was reacted via thiol-ene coupling with olive, rice bran, grape seed, and linseed oils for the synthesis of polyols and the corresponding polyurethane films could be prepared with tunable thermomechanical properties for application in coatings, synthetic leathers, and adhesives [67]. Another possible method is the ring-opening reaction of epoxidized soybean oil with lactic acid (Figure 9), which was used together with oligomeric diphenylmethane diisocyanate to prepare rigid polyurethane foams [68].

The environmental concern of the whole process is also a major challenge regarding polyurethanes, and a cradle-to-gate life cycle assessment for a pilot-scale polyol production from rapeseed oil was recently reported [69]. Research has also focused on preparing vegetable oil-based non-volatile polyisocyanates, thus combining safety and renewable resources [6]. A thermo-reversible polyurethane network based on vegetable oils was prepared by reacting a furan oligomer derived from oleic acid, a linear rapeseed oil-based polyurethane, and a bismaleimide via the Diels–Alder reaction [70]. More complex systems with specific applications have also been considered, such as vegetable oil-based polyurethanes for tissue engineering [71]. In this case, thermosets with good cytocompatibility and efficient antimicrobial activity against various microbial strains may be prepared and considered for wound dressing applications. Non-isocyanate polyurethanes based on vegetable oils also represent a significant step towards more sustainable materials [72].

An additional green connotation was added to vegetable oil-thermosets by associating monomers prepared from high-oleic soybean oil and lignin. Azide-functionalized soybean oil polymers were prepared and submitted to click crosslinking with alkyne-modified lignin via a metal-free thermal azide-alkyne cycloaddition reaction, leading to the preparation of elastomers consisting of a network of long flexible polymer chains [73]. The three-dimensional character of the final materials in this particular study was provided by the polyfunctional nature of lignin, as the triglyceride itself was converted, in a first step, to its corresponding fatty esters by transesterification with 2-(methylamino)ethanol.

In some cases, the introduction of fatty acid derivatives into macromolecular chains can not only be a sustainable alternative but also improve some properties and widen the possible applications of final materials. Starch, for instance, is a highly hydrophilic macromolecule, and is hence sensitive to humidity and with poor miscibility with hydrophobic materials. A recent study performed a biocatalyzed esterification reaction between fatty acids from high oleic vegetable oils and potato starch, giving hydrophobic starch derivatives [17]. Other examples consider that unsaturated fatty acid chains can form crosslinked structures. In a recent publication, the authors copolymerized soybean methacrylate with either styrene, methyl methacrylate, or butyl acrylate. The double bonds from the soybean oil-based comonomer underwent auto-oxidation and formed crosslinked structures with drastically enhanced mechanical properties, viz., good candidates for coating applications [74]. A more sustainable approach copolymerized soybean methacrylate with cardanol methacrylate, being the first latex produced with fully plant-based comonomers to be reported in the literature [75]. Another recent contribution performed a physical crosslinking between copolymers of soybean methacrylate and 2-carboxy methyl acrylate by metal-ligand coordination [76].

Another aspect worth mentioning is the utilization of vegetable oils and derivatives not as precursors to macromolecules, but as additives. In a recent study, rubbery materials were developed by grafting renewable fatty acids onto epoxidized soybean oil. These rubbers acted as tougheners for thermosetting epoxy resins [77]. In another investigation, neem oil was epoxidized and added as a plasticizer to rubbers, improving or maintaining comparable mechanical properties with respect to rubbers containing traditional plasticizers. The authors concluded that epoxidized neem oil may serve as a cheap and sustainable replacement for traditional petroleum-based rubber plasticizers [78]. Acrylates containing a triazine ring as a rigid core and long fatty acid chains as soft arms were synthesized and added to acrylated epoxidized soybean oil to produce UV-curable coatings with improved thermal and mechanical properties [79].

As it can be inferred from the many different possible strategies that may be carried out, the use of pristine and chemically modified triglycerides as monomers that possess their multifunctional nature preserved is an important strategy for the preparation of original crosslinked materials with lower T_g_, with respect to other commercial resins based on the fossil platform. A multitude of different works on the topic is continuously being reported in the literature, which is a significant indicator of a promising approach. However, considering the utilization of vegetable oils as a platform for the design of original macromolecular materials on a large scale, hydrolysis and transesterification reactions are more frequently conducted for the preparation of easily processable thermoplastic materials, and the most relevant and recent contributions discussed below.

## 4. Linear Polymers Based on Fatty Acids or Their Ensuing Derivatives

**Figure 10:**
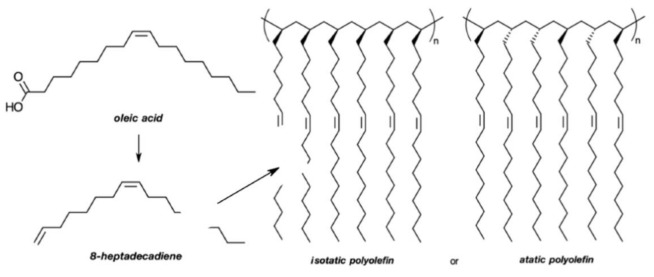
Schematic representation of the conversion of oleic acid into polyolefins. Adapted from Ref. [82].

The concept of producing linear polymers from plant oils has changed drastically in the past 70 years. The discreet example of Rilsan^®^ has evolved into the utilization of vegetable oils as the main components of sophisticated materials based on synthetic processes for the design of polymers with very specific applications [6]. The market for polyolefins is increasing yearly, which motivates the search for novel sources of monomers and polymers with original properties, as emphasized by very recent research. Lomège and coworkers published a very complete review covering the possible routes to convert fatty-acid-based monomers into polymers via radical polymerization [13]. This monograph brings to light many examples of polyolefins that can be easily prepared from vegetable oils, with the most diverse type of pendant groups along the polymer chain. Another recent contribution is related to the preparation of ABA triblock copolyolefins from lauryl acrylate and triacetic acid lactone, leading to materials that are suitable for high-performance pressure-sensitive adhesives [80]. Isomerizing copolymerization was used to copolymerize high-oleic oils or fatty acid methyl esters with ethylene in a one-pot reaction [81]. The isolated polyolefins had Mn values higher than 30 kDa, comprising more than 20 wt.% of plant oils. A very interesting study recently reported the synthesis of 8-heptadecadiene from oleic acid, and its subsequent polymerization using zirconocene complexes with methylaluminoxane catalysts, which produced isotactic and atactic polyolefins (Figure 10). The unsaturated pendant groups allowed the conduction of a further step of post-polymerization crosslinking [82], as well as post-polymerization chemical modifications, such as bromination, epoxidation, and cross-metathesis [83].

The catalytic conversion of unsaturated fatty acids by isomerizing carbonylation or olefin metathesis yields (ultra)long-chain AB-type monomers bearing terminal dicarboxyl, diol, or diamine groups [84]. These vegetable oil-based monomers can be polymerized to polyesters, polycarbonates, and other (ultra)long-chain polycondensates, corresponding to polyethylene-like materials with an additional non-persistent feature. In fact, the synthesis of novel linear polyesters based on the vegetable oil platform is of great interest, with many promising contributions available in the literature. A monomer derived from castor oil obtained through tiol-ene click chemistry was copolymerized with another castor oil-based monomer, obtaining poly(β-thioether ester-co-ricinoleic acid), a copolymer that is degradable under acidic conditions [85]. A polyester from dimer acid from soybean oil and polyethylene glycol was synthesized and showed significantly better biodegradation compared to terephthalic acid-based polyesters [86]. The castor oil-derived undecenol was submitted to catalytic functionalization/polymerization, yielding an aliphatic polyester in a single-step procedure under mild conditions [87]. Unsaturated polyesters were prepared through cross-metathesis of oleic acid and oleic alcohol [88], and elastomeric copolyesters were synthesized from fatty acid diol monomers obtained from the reaction of stearic, oleic, linoleic acid, and 7-hydroxy-4-methyl coumarin with diethanolamine [89]. Linear polyesters from oleic acid-based monomers were prepared and later converted into polyurethanes [88]. Polyesters prepared from microalgae oil lipids have also been described [90].

Less frequent, but also of great interest, is the design of polyamides and polycarbonates from vegetable oils. A series of polyamide 11 copolymers was synthesized via melt polycondensation from 11-aminoundecanoic acid, hexamethylenediamine, and a dimer acid obtained from unsaturated fatty acids through an acid-catalyzed Diels–Alder reaction [91]. The thermal and mechanical properties could be tuned by increasing the amount of dimer acid incorporated into the polyamide, as it increased the chain irregularity and therefore reduced the hydrogen bonding strength of the amide groups. In another publication, linear polyamide films from an unsaturated diester of canola oil, dimethyl 9-octadecenedioate, and p-xylenediamine or diethylenetriamine were synthesized and characterized. The presence of unsaturations in the polymer backbone allowed postpolymerization functionalizations to insert side chains and tune the final properties of the materials [92]. A very interesting approach for the synthesis of polycarbonates was reported by Cramail’s group, based on the controlled polymerization of a fatty acid-based cyclic carbonate-bearing an unsaturation. The ensuing linear polymer was grafted with thio-cinnamate, a photo-sensitive moiety that allowed the material to form crosslinks when irradiated with a UV light at 365 nm, and to return to its linear form when the wavelength was reduced to 254 nm [93]. The preparation of polycarbonates is particularly advantageous when carbon dioxide is used as raw material, and this strategy was reviewed considering also the utilization of other biobased feedstocks, such as plant oils, glycerol, and fatty acids [94]. Vegetable oil-based fatty dimer diamines with long alkyl branches (also referred to as Priamine) are interesting building blocks that may be used for the synthesis of polyamides, polyimides, polyurethanes, and poly(urethane-urea)s by polycondensation [95,96,97]. Historically, the general idea of producing thermoplastic polymers from vegetable oils transcended quickly the laboratory scale and reached industrial production at the very beginning of the second half of the XX century. This fact is per se a confirmation of a very promising strategy in terms of an economically viable process based on green chemistry, which has been gaining growing attention over the last few years. It is expected, therefore, that sophisticated thermoplastic materials based on plant oils may reach the market progressively, once it is aligned with the concept of sustainable development.

## 5. Vegetable Oils-Based Composite Materials

The multitude of interesting polymeric materials that can be prepared from the vegetable oil platform has allowed the widening of properties that can be obtained by associating them with fibers and other fillers to achieve materials of improved physical, thermal, and mechanical properties. Zhang and coworkers tackled the broad field of polymers and composites based on triglycerides and their derivatives [22]. As for composites, the authors focused on materials comprising disperse phases of clay, carbon nanotubes, and graphene, metals and silica oxide, synthetic fibers such as glass and carbon fibers, lignocellulosic fibers, and lignin. Some years after the publication of this very complete and informative monograph, many other related studies were conducted, as discussed below.

A thorough search for the most recent contributions on the preparation of composites from vegetable oils leads, mostly, to polyurethanes reinforced with natural fibers, which is in accordance with the growing interest for materials of reduced environmental impact. Castor oil is often considered for the synthesis of polyurethanes, as it naturally bears three hydroxyl groups per triglyceride, which promptly react with isocyanates. On the other hand, isophorone diisocyanate, a cycloaliphatic diisocyanate, has been widely used for the synthesis of aqueous dispersible polyurethanes (or waterborne polyurethanes, WPU), since it provides exceptional weathering resistance for the materials. The most original strategies consider chemically modified fibers and/or slight variations on the formulations of the matrix, including other naturally available compounds, to increase the green connotation of the final product. Hormaiztegui, Mucci and Aranguren [98] developed WPUs from castor oil, polycaprolactone diol, isophorone diisocyanate and tartartaric acid. Composite materials were prepared by mixing different proportions of two aqueous suspensions, the first containing the WPUs and the other cellulose nanocrystals, prepared by acid hydrolysis of commercial microcrystalline cellulose powder. The materials exhibited increased glass transition temperature, storage modulus, and thermal stability, and, as expected, an improved ability to maintain integrity when immersed in water. Castor oil and isophorone diisocyanate were also applied to the synthesis of WPUs, further reinforced with TEMPO-oxidized cellulose microfibers, to prepare composites with improved tensile strength and thermal stability [99]. TEMPO cellulose was synthesized from bleached paperboard and functionalized with octa(aminopropyl) polyhedral oligomeric silsesquioxane [100]. The fibers were then incorporated into a WPU matrix of castor oil and isophorone diisocyanate. The results showed improved mechanical properties, thermal stability, and water resistance of the composite films. Other natural macromolecules have also been considered as disperse phase, in a strategy that can be illustrated by the work of Wang and collaborators [101], describing the association of castor oil, 2,2-dimethylolbutanoic acid, and isophorone diisocyanate for the preparation of WPUs that were mixed with sodium alginate to produce composites with tunable properties. Moreover, different amounts of sodium lignosulfonate were incorporated into castor oil-based WPUs, leading to materials that may be used as UV absorption coatings and applied in the formulation of sunscreen creams [102]. An interesting association between castor oil-based WPUs and carboxymethyl chitosan was considered for the preparation of novel composites [103]. For this purpose, predetermined amounts of a carboxymethyl chitosan aqueous solution were introduced into WPU dispersions under mechanical stirring, and the composite films were prepared by casting. The authors observed the formation of semi-interpenetrating polymer networks and a high degree of hydrogen bonding between the phases, which resulted in an increase of the hard segment contents and, therefore, superior mechanical performances.

Composites with matrixes comprising polyurethanes prepared with hydroxylated vegetable oils and 4,4′-methylene diphenyl diisocyanate (MDI) or toluene diisocyanate (TDI) have also been reported. Nanocomposite biofoams were synthesized from a soybean phosphate ester polyol (prepared from fully epoxidized soybean oil and phosphoric acid), Jeffol A-630, and oligomeric MDI (pMDI) [104]. In this case, hydroxyl-functionalized multiwalled carbon nanotubes were incorporated to confer high conductivity, thermal insulation, and low density. Natural fibers are, however, the preferred choice, and a thorough analysis of the effect of the incorporation of different cellulose nanowhisker polymorphs as reinforcement of jatropha oil-based polyurethanes was recently published [105]. The strategy has an additional green connotation when residues of vegetable oil industries may be valorized, which was the case of the preparation of rigid polyurethane-polyisocyanurates foams containing 30–60% of milled rapeseed cake, a by-product of the rapeseed oil production [106].

Vinyl resins are also an interesting choice of polymers that can be prepared from vegetable oils, as many of their petroleum-based counterparts and ensuing composites find important industrial applications because of their excellent thermal and mechanical properties, such as high glass transition temperatures, strengths, thermal stability, and corrosion resistance [22]. To overcome the low reactivity of crude vegetable oils towards addition polymerization, many authors reported, for example, the use of preliminary steps of isomerization or (metha)acrylation of non-conjugated vegetable oils to convert them into reactive monomers for polymer synthesis. The development of original composites can be largely beneficiated from this approach, but as the degree of sustainability of the final material must be increased, and the renewable character of both continuous and disperse phases is required, one can often come across issues of incompatibility between them. Chen and Liu [107] reported the preparation of vegetable oil-based vinyl composites using an intelligent strategy, based on the chemical modification of microcrystalline cellulose through the esterification of the hydroxyl groups of the fibers’ surface with methacrylic anhydride. Then, the functionalized fibers were used as reactive reinforcing agents for crosslinking with acrylated epoxidized soybean oil using 2 wt.% of tert-butyl peroxybenzoate as initiator. The authors stated that the incorporation of unsaturations along the cellulosic fibers and their utilization as a reactive reinforcing agent for acrylated epoxidized soybean oil resins induced the formation of co-continuous composites with improved flexural strength and modulus, storage modulus, glass transition, thermal stability, and water resistance.

Vegetable oil-based epoxy resins can be prepared by the direct polymerization of epoxidized vegetable oils, as well as by their polymerization with amines, carboxylic acids, and anhydrides as curing agents. However, their low glass transition temperatures resulting from the low crosslinking density of the networks limits further applications to nonstructural devices, coatings, and additives [22,108], and the incorporation of a disperse phase for the design of composite materials may overcome this limitation. Varying amounts of epoxidized waste vegetable oils and the diglycidyl ether of bisphenol-A were used to prepare epoxy resins, which were applied as matrixes in composites reinforced with up to 30% of carbon fibers [109]. More recently, epoxidized soybean oil was reacted with 9,10-dihydro-9-oxa-10-phosphaphenanthrene-10-oxide and the ensuing epoxy resins were associated with rice husk silica [110]. The authors examined the influence of the additives on the mechanical properties and flame-retardant capabilities of the materials, demonstrating a better performance in the case of the composites. A few studies have reported the preparation of vegetable oil-based-epoxy resin using bio-based curing agents, and their application as a matrix in composites with natural or synthetic fibers was recently reviewed [108].

The surface functionalization of lignocellulosic fibers with vegetable oils is a sustainable and useful option for compatibilization between phases when fewer polar matrixes are used. Cellulose pulp was chemically modified with maleinized high-oleic sunflower oil and dispersed in an unsaturated polyester matrix [111]. The composites bearing functionalized cellulose exhibited improved tensile strength, compressive strength, and elongation compared with those produced with the untreated cellulose. Vegetable oils may also serve as sustainable plasticizers, and a few examples may be cited, mainly based on maleinized oils [112,113,114,115,116].

Although not detailed in this section, the association between vegetable oil-based polymers and other inorganic fillers (such as clay and metals) is very promising [117]. Diez-Pascual [118] published a monograph tackling the development of antibacterial nanocomposites based on polymers derived from vegetable oils and nanoparticles of TiO_2_, ZnO, CuO and, Fe_3_O_4_. The design of original multiphase materials based on plant oils is promising, mainly considering disperse phases of renewable origin as well. They often present excellent mechanical performances together with other desirable properties that are suitable for coatings, paints, adhesives, and food packaging, among other applications.

## 6. The Case of Tung Oil: An Old Ally for Original Materials

In the last decades, due to its low price, large availability and peculiar chemical structure, the large exploitation of tung oil in different contexts can be considered a strong indication of its high potential to act as a rich source of molecules for the production of energy and materials. As a non-edible vegetable oil, it may be easily incorporated into the biodiesel industry without impacting the food and feed supply chain, leading to fatty acid methyl esters that meet the ASTM quality standards of kinematic viscosity at 40 °C, density at 25 °C, cloud point and pour point [119]. The use of tung oil for the synthesis of polymers is, however, much more promising in terms of the possibilities to develop smart functional materials that can be useful for a wide domain of applications.

The most classic attribution of tung oil, referring to centuries ago when men first coated wooden objects to improve their properties and to waterproof ships, is still an object of study. Researchers closely assessed the benefits of incorporating tung oil for the treatment of wood, which has led to materials of improved dimensional stability, moisture absorption, thermal stability, and morphological characteristics, which has encouraged its implementation in buildings, furniture, and landscape architecture to broadly expand the application of wood products [120]. In this and other related cases, the ease with which tung oil is converted to a surface-dry film is given by its prompt susceptibility to the radical oxydopolymerization reaction, a process that cannot be avoided in the presence of atmospheric oxygen. In fact, due to the possible formation of stable conjugated radical intermediates, tung oil can be homo- and copolymerized, in a strategy that was first described in the late 1960s and again in the early 1980s [121]. The most recent contributions consider a prior chemical modification of tung oil with acrylic and methacrylic moieties, leading to materials of superior thermal and mechanical properties, which are readily cured by UV light [122,123]. A more original strategy called upon the copolymerization reaction of crude tung oil and furfuryl methacrylate under inert atmosphere, initiated by benzoyl peroxide, and the final networks were obtained in a second step based on the Diels–Alder click reaction between pendant furan moieties and residual conjugated double bonds of tung oil, which was performed under high pressure and high temperature [124]. The same acrylated tung oil was associated with styrene, and the copolymerization reaction was initiated by tert-butyl peroxybenzoate [125]. Herein, the authors used this polymer network as the continuous phase in composites reinforced with nanocellulose from onion skins, and the final materials exhibited significantly improved storage modulus, strength (a 190- to 240-fold increase in mechanical strength), and increased hydrophobicity. In fact, in the last couple of years, tung oil and its derivatives have been more frequently explored for the preparation of composites. Murawski and Quirino [126] reported an interesting strategy for the design of original composites made of tung oil-based resins reinforced with cellulose fibers. N-butyl methacrylate was vortexed with crude tung oil, or its main fatty acid (α-eleostearic acid), or its corresponding fatty acid methyl ester (methyl α-eleostearate), α-cellulose, and di-*tert*-butyl peroxide prior to cure in a convection oven, and the final properties of the crosslinked materials were compared, with the purpose of preparing composites with enhanced resin-reinforcement interactions. The authors concluded that the composite prepared with α-eleostearic acid exhibited more favorable interactions with water due to its higher polarity. On the other hand, thermo-mechanical analyses indicated that the tung oil resin had superior properties due to its higher crosslink density. Other composites based on the tung oil platform that are worth mentioning were prepared from the reaction of a tung oil monoglyceride and pyromellitic anhydride to obtain alkyd resins, which were further reacted with diaminodiphenyl sulfone [127]. This continuous phase was reinforced with polypyrrole-enveloped polysorbate modified cerium oxide nanoparticles, leading to nanocomposite coatings with corrosion protective performances. Another example of a hybrid inorganic-organic material was reported by the same authors, comprising a hydrophobic bi-functional ceria doped tung oil polyesteramide, which exhibited corrosion protectiveness and flame retardancy [128].

**Figure 11:**
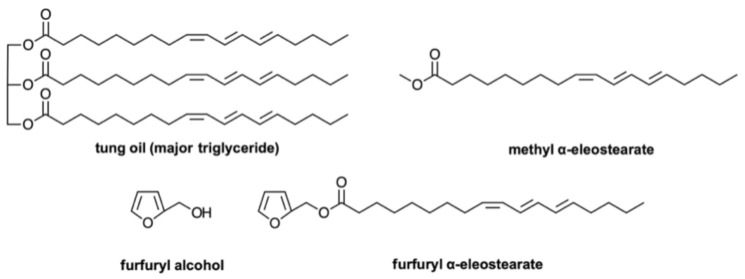
Monomers used for the preparation of tung oil/furfuryl alcohol networks. Reproduced with permission from Ref. [132]. Copyright International Journal of Biological Macromolecules 2020.

The cationic homo- and copolymerization of tung oil is sporadically explored in the literature, in a strategy that was first reported by Larock’s group in 2000 [121] and followed by a series of related works aiming to convert tung oil into three-dimensional networks, mostly using boron trifluoride diethyl etherate as initiator together with comonomers such as styrene, divinylbenzene, methyl α-eleostearate, terpenes and poly(caprolactone) [2,6]. Nanoscale semi-interpenetrating polymer networks of poly(ε-caprolactone) and polymerized tung oil were prepared via in situ cationic polymerization and compatibilization in a homogeneous solution [129]. The materials were characterized by FTIR, rheological measurements, dynamic mechanical analysis (DMA), and morphological features, which varied largely with respect to the PCL content. The isothermal and non-isothermal crystallization kinetics of the materials were also evaluated [130]. A study of the formulation effect of cationically polymerized vegetable oil thermosets made of acrylated epoxidized soybean oil, methyl α-eleostearate, styrene, and divinylbenzene, was recently reported [131]. In all cases, no detailed mechanisms for the processes were put forward and most studies were carried out in an atmospheric environment and called upon the use of high temperatures for the final setting phase, so that, inevitably, free radicals also intervened in the network formation. Given the lack of information on the cationic polymerization mechanism of tung oil, an investigation on this process was conducted using trifluoroacetic acid (TFA) as initiator, room temperature, and an inert atmosphere [132], and although some insight could be gathered from the tung oil polymerization, the rapid formation of crosslinked materials hindered a deeper understanding of the mechanistic and structural issues. The methyl ester of α-eleostearic acid was therefore selected to mimic tung oil in this context because its cationic polymerization proceeded without leading to crosslinked products, thus allowing its progress to be followed in a homogeneous medium, notably by NMR spectroscopy [132]. The idea evolved into a more complex system, based on the cationic copolymerization of furfuryl alcohol with tung oil, in order to combine the high intrinsic flexibility of the crosslinked tung oil polymers with the stiffness of the furfuryl alcohol resin, leading to fully biobased crosslinked materials with a tunable glass transition temperature [133]. Three approaches were investigated, also using TFA as initiator, viz. (i) combining crude tung oil and furfuryl alcohol, (ii) combining methyl α-eleostearate and furfuryl alcohol, and (iii) polymerizing furfuryl α-eleostearate as a single monomer from which a furfuryl/triene interpenetrating network could be generated (Figure 11). The polymerization reactions with varying TFA concentrations were followed by NMR spectroscopy, and it was possible to get valuable information on mechanistic aspects and product properties.

**Figure 12:**
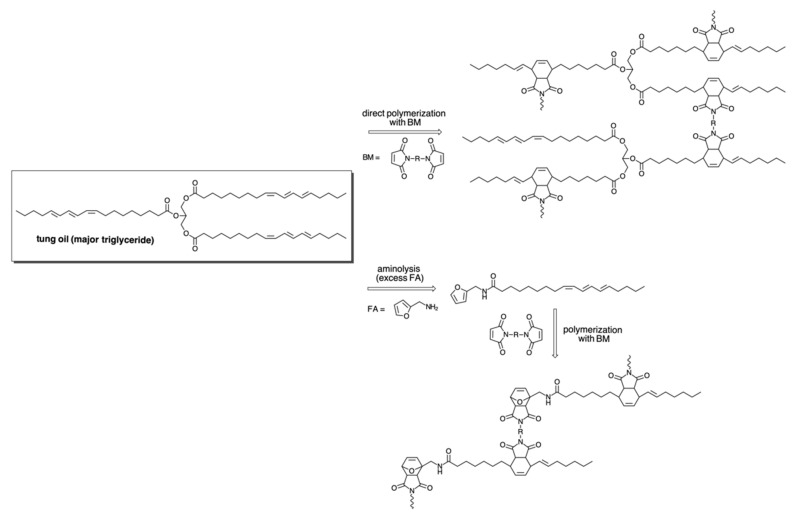
Schematic representation of the use of tung oil to produce Diels–Alder-based polymers. Adapted from Ref. [134] with permission from The Royal Society of Chemistry.

Step-growth polymerization is also possible for the design of original tung oil-based polymers [6], and in this case, the prompt reactivity of the three conjugated double bonds of α-eleostearic acid towards dienophiles in Diels–Alder click reactions are often explored to achieve this goal. Tung oil was submitted to two different reactions, viz. (i) its direct polymerization with bismaleimides for the synthesis of networks, and (ii) its bulk reaction with an excess of furfurylamine, leading to the formation of an AB monomer containing a furan ring and the three conjugated double bonds of α-eleostearic acid, to be further polymerized with bismaleimides [134] (Figure 12). More recently, the Diels–Alder reaction of tung oil with 4-maleimidophenol was conducted for the synthesis of polyols and, in a further step, of polyurethane elastomers [135]. Eleostearic diethanolamide was synthesized from tung oil and diethanolamine and then reacted with hydroxymethylmaleimide to give a triol, which was mixed with castor oil and isophorone diisocyanate for the synthesis of polyurethanes [136].

**Figure 13:**
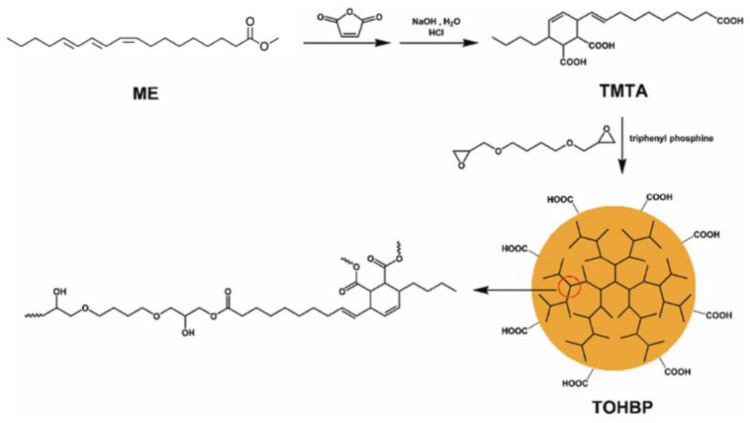
Synthesis of hyperbranched polymers from methyl α-eleostearate and maleic anhydride. Reproduced with the permission from Ref. [138]. Copyright New Journal of Chemistry 2020.

The malenization reaction of vegetable oils is also a very important strategy that has been used for the synthesis of macromolecular materials [6]. Tung oil has indeed been submitted to such reactions, as in the case of the interesting work reported by Zhang et al. [137], in which tung oil was reacted with maleic anhydride via a Diels–Alder mechanism, and the product was further reacted with citric acid by a ring-opening reaction assisted by microwave. The oligomers were then reacted with glycidyl methacrylate, again by ring-opening, leading to UV-cured materials. In the same context, hyperbranched polymers were synthesized from a tung-maleic tricarboxylic acid, prepared via the reaction between methyl α-eleostearate and 1,4-butanediol diglycidyl ether [138] (Figure 13). The hyperbranched polymer was then used to prepare epoxy resins by the reaction with diglycidyl ether of bisphenol A (DGEBA) and a commercial polyetherdiamine. Thermal, mechanical and morphological analysis showed interesting results, and the authors considered that the material may be very promising to replace petroleum-based epoxy resins in the future.

**Figure 14:**
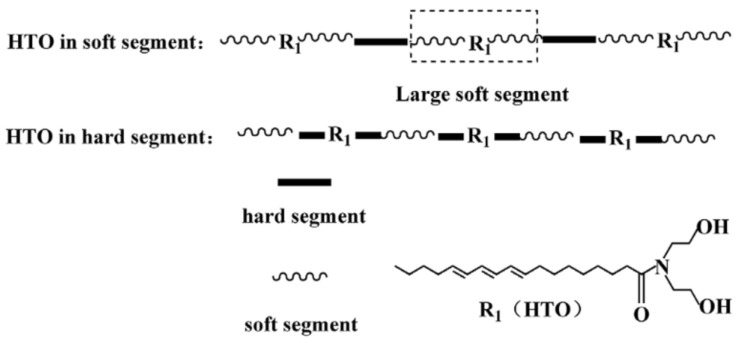
Schematic representation of the HTO-based WPU dispersions. Reproduced with the permission from Ref. [139]. Copyright Journal of Applied Polymer Science 2020.

Tung oil has also been used for the synthesis of waterborne polyurethanes (WPU). Hydroxylated tung oil (HTO) was prepared by ester aminolysis via the reaction of tung oil and diethanolamine [139]. WPUs were then obtained by reacting it with polytetramethylene ether glycol, dicyclohexylmethane diisocyanate or isophorone diisocyanate. The originality reported by the authors is the possibility of positioning the hydroxylated tung oil at controlled locations in the polymeric chain (Figure 14), which is a useful strategy to tune the final properties of the materials.

Cationic WPUs were developed by, firstly, reacting methyl α-eleostearate and diethanolamine, which gave a tung oil-based diol, susceptible to react with poly(ethylene glycol) and isophorone diisocyanate to give an NCO-terminated polyurethane prepolymer [140]. A further reaction with diethanolamine was conducted to produce an aminated polyurethane, that was cationated with hydrochloric acid. The materials exhibited good activity against *Escherichia coli*, indicating a good potential as, for example, antimicrobial coating materials. Recently, the design of tung oil-loaded polyurethane microcapsules covered with a double-layer shell of polyaniline was reported, and self-healing anti-corrosion coatings were prepared by adding 10 wt.% of these microcapsules into an epoxy resin [141].

The important number of investigations to convert tung oil into original macromolecular materials bodes well for the expectation of seeing it applied on a large scale in the near future, not only for the protection of wood products but also to serve as a platform for more sophisticated materials.

## 7. Conclusions

The exploitation of vegetable oils has evolved radically during the last decades, and modern processes are now available to convert them into energy and materials. The latter strategy is particularly impressive, with promising ongoing research and development initiatives, indicating that this widely available renewable resource can now provide the basic building blocks of original and high-tech macromolecular materials. The current scenario is expected to expand further, with plant oil-based polymers appearing more frequently as sources of commercial products capable of replacing important counterparts prepared from the fossil platform.

## Figures and Tables

**Table 1:** Major vegetable oils: world supply and distribution (commodity view) (million metric tons). Data from the United States Department of Agriculture (USDA) [25].

Oilseeds	2002/2003	2019/2020		
	Production	Production	Imports	Exports
Cottonseed	3.51	44.42	0.66	0.84
Palm kernel	3.36	19.38	0.11	0.08
Peanut	4.62	46.06	4.36	4.69
Rapeseed	12.21	69.22	15.55	15.53
Soybean	30.57	336.46	165.43	165.17
Sunflower seed	8.12	54.89	3.27	3.58
Total	62.39	570.43	189.37	189.88

**Table 2:** Iodine values of some unsaturated fatty acids and their triglycerides. Reproduced with permission from Ref. [6]. Copyright John Wiley & Sons and Scrivener Publishing 2019.

Fatty Acid (Number of Carbon Atoms)	Iodine Value	
	Acid	Triglyceride
Palmitoleic (C16)	99.8	95
Oleic (C18)	89.9	86
Linoleic (C18)	181	173.2
Linolenic (C18) and α-eleostearic (C18)	273.5	261.6
Ricinoleic (C18)	85.1	81.6
Licanic (C18)	261	258.6

## Data Availability

The study did not report any new data.
